# Minimally invasive direct coronary bypass compared with percutaneous coronary intervention for left anterior descending artery disease: a meta-analysis

**DOI:** 10.1186/s13019-016-0512-1

**Published:** 2016-08-05

**Authors:** Xiao-Wen Wang, Can Qu, Chun Huang, Xiao-Yong Xiang, Zhi-Qian Lu

**Affiliations:** 1 Department of Cardiothoracic Surgery, The First Affiliated Hospital of Chongqing Medical University, Chongqing, 400016 People’s Republic of China; 2 Department of Cardiothoracic Surgery, Shanghai Jiao Tong University Affiliated Sixth People’s Hospital, Shanghai, 200233 People’s Republic of China; 3 Department of Pharmacy, The First Affiliated Hospital of Chongqing Medical University, Chongqing, 400016 People’s Republic of China

**Keywords:** Left anterior descending coronary artery, Minimally invasive direct coronary artery bypass, Percutaneous coronary intervention, Outcome

## Abstract

**Background:**

The clinical outcomes for left anterior descending (LAD) coronary artery lesion between minimally invasive direct coronary artery bypass (MIDCAB) and percutaneous coronary intervention (PCI) are still controversial. The objective was to compare safety and efficacy between MIDCAB and PCI for LAD.

**Methods:**

Electronic databases and article references were systematically searched to access relevant studies. End points included mortality, myocardial infarction, target vessel revascularization (TVR), major adverse coronary events (MACE), angina recurrence, and stroke.

**Results:**

Fourteen studies with 941 patients were finally involved in the present study. The mortality and incidence of myocardial infarction were similar in MIDCAB and PCI groups at 30 days, 6 months, and at follow-up beyond 1 year. Compared with PCI, MIDCAB decreased incidence of TVR and MACE at 6 months and beyond 1 year follow-up. MIDCAB was associated with a lower incidence of angina recurrence at 6 months compared with PCI. PCI was associated with higher risk of restenosis in target vessel. No significant difference was shown for stroke.

**Conclusion:**

Our meta-analysis indicates that there are no significant differences in the safety between MIDCAB and PCI in patients with LAD. However MIDCAB is superior to PCI for TVR and MACE.

## Background

Minimally invasive direct coronary artery bypass (MIDCAB) and percutaneous coronary intervention (PCI) are alternative procedures to mechanical revascularization for patients with left anterior descending (LAD) coronary artery lesion. The effects of these two procedures on cardiovascular outcomes have been compared in several clinical trials. Further, the comparative effectiveness of MIDCAB and PCI remains poorly understood for patients in whom both procedures are technically feasible and coronary revascularization is clinically indicated. Since the publication of those meta-analyses, additional trials and long term results have become available Meta-analyses of individual patient data from clinical controlled trials substantially have the potential to increase the power and improve the precision of treatment effects and safety. Thus, the goal of this study was to perform a meta-analysis of trials to evaluate the comparative effectiveness of MIDCAB and PCI among patients with isolated lesions of the LAD.

## Methods

### Search strategy and selection criteria

Two investigators (WX and QC) independently searched the literatures collected in PubMed, MEDLINE, EMBASE, Science Direct, ISI Web of Knowledge, and Cochrane databases up to August 1, 2015. Search terms included: percutaneous coronary intervention, stent, minimally invasive coronary artery bypass, MIDCAB, off-pump coronary surgery, left anterior descending, and clinical trial. We also sought additional studies by reviewing the reference lists of included articles, conference abstracts, and the bibliographies of expert advisors. The searches were limited to English publications in humans. We did not include abstracts or meeting proceedings. This search strategy was performed iteratively until no new potential citations could be found on review of the reference lists of retrieved articles.

Studies were included if they met all of the following criteria: (1) studies comparing MIDCAB with PCI for the treatment of isolated lesions of LAD; (2) reporting at least one pertinent clinical outcome. Exclusion criteria included: (1) duplicate publication, (2) ongoing/unpublished study, (3) studies published only as an abstract or in conference proceedings. Hybrid and robotically assisted surgery studies were excluded. In addition, if the same author published multiple studies reporting outcomes at different follow-up points, we extracted patient characteristics from the first study, with data for outcomes of interest at subsequent follow-up times extracted from the later studies. When two studies by the same institution reported the same out comes at similar follow-up periods, we included in our analysis either the better quality or the most informative publication.

### Data extraction

The quality of the studies was assessed by using the Newcastle Ottawa Scale system, in particular the use of stars awarded for each numbered criterion item. Two of us (WX and QC) evaluated the quality of all included studies by examining three items: patient selection, comparability of MIDCAB and PCI groups, and assessment of outcomes.

All data were extracted from article texts, tables, and figures. Two individual investigators (WX and QC) independently extracted data on patient and study characteristics, outcomes, and study quality for each trial using a standardized protocol and reporting form. Disagreements were resolved by consensus with a third reviewer (LZ).

### Study outcomes and definitions

The end points of this meta-analysis were as follow: (1) mortality; (2) major adverse coronary events (MACE); (3) target vessel revascularization (TVR); (4) myocardial infarction (MI); (5) angina recurrence; (6) restenosis; (7) stroke. Death was defined as death from any cause. The original study authors’ definition of MACE was used and usually included the composite of death, myocardial infarction, and cerebrovascular accident or stroke. TVR was defined as repeat PCI of the treated vessel including any segment of the left anterior descending and/or left circumflex coronary artery. MI included Q-wave MI and non-Q-wave MI. Agina recurrence and restenosis were defined according to study authors’ definitions. Stroke was defined as a sudden neurologic deficit resulting from vascular lesions of the brain including hemorrhage, embolism, or thrombosis.

### Statistical methods

We used fixed-effects or random-effects models to produce across-study summary relative risk (RR) with 95 % confidence interval (CI). The pooled effects were calculated using fixed-effect model with the Mantel-Haenszel method when there was no significant heterogeneity or with DerSimonian–Laird weights for the random effects model when there was significant heterogeneity. The chi-square test was used to study heterogeneity between trials, and the I^2^ statistic was used to estimate the percentage of total variation across studies. I^2^ value greater than 50 % was considered as significant heterogeneity. Sensitivity analyses were performed to compare the treatment effects obtained from different subgroups with the overall treatment effects. Publication bias was explored through visual inspection of funnel plots and assessed by applying the Egger weighted regression statistic with a p value < 0.05 indicating significant publication bias among the included studies. Correction for publication bias was performed using trim-and-fill methods. A p value < 0.05 was regarded as significant. All statistical analyses were performed using Review Manager (version 5, Cochrane Collaboration, Oxford, UK).

## Results

### Characteristics of included studies

**Fig. 1:**
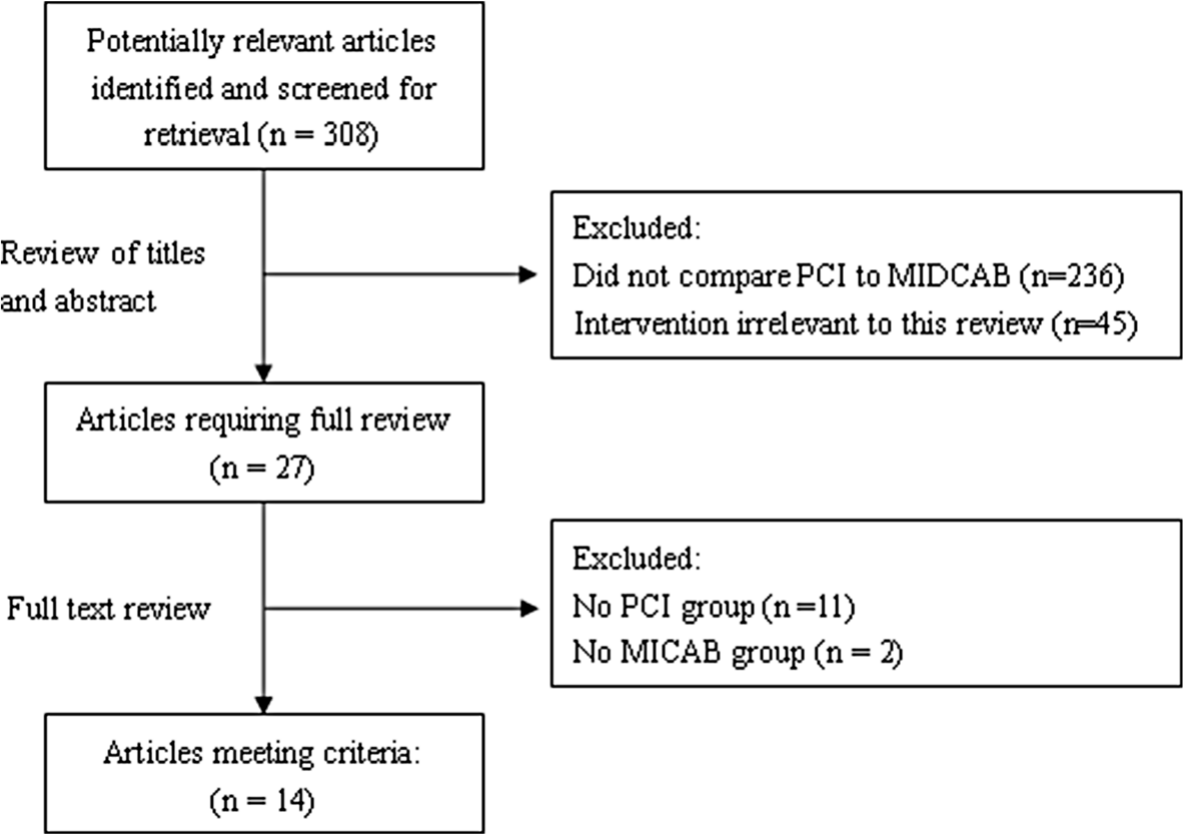
Flow diagram of studies included in the final meta-analysis

The literature search identified 308 relative references. After selection according to the inclusion/exclusion criteria, 14 studies were eligible for meta-analysis finally (Fig. 1). A total of 941 patients were involved, of whom 495 patients undergoing PCI and 446 patients undergoing MIDCAB, as summarized in Table 1 [1–14]. Two groups each published three studies reporting on the same patient group but were included due to they reported outcomes at different follow-up periods in each of these studies.

**Table 1:** Summary of baseline patient characteristics from comparative studies included in the present meta-analysis

	No. of patients		Age (years)		Male (%)		Hypertension (%)		Diabetes (%)		Previous cerebrovascularevent	
Author	PCI	MIDCAB	PCI	MIDCAB	PCI	MIDCAB	PCI	MIDCAB	PCI	MIDCAB	PCI	MIDCAB
Cisowski [1–3]	50	50	53.3 ± 10.2	54.1 ± 9.1	42	41	26 (52 %)	28 (56 %)	4 (8 %)	3 (6 %)	NR	NR
Drenth [3, 5–7]	51	51	61 (1.3)	60 (1.6)	75	78	33	16	18	24	NR	NR
Diegeler [8–10]	110	110	62.5 ± 10.2	61.6 ± 10.0	72	77	72	71	34	25	NR	NR
Reeves [11]	50	50	54.5 (49-61)	58.8 (53–67)	86	70	NR	NR	NR	NR	NR	NR
Hong [12]	119	70	60.5 ± 9.6	61.4 ± 9.9	63.9	64.3	50.4	55.7	37	48.6	2.5	2.9
Kim [13]	50	50	61 ± 12	63 ± 12	60	70	55	55	20	15	2	2
Thiele [14]	65	65	66 (59–72)	66 (59–71)	69	71	83	85	28	25	3	9
	Previous MI (%)		Smokers (%)		Hypercholesterolaemia (%)		Ejection fraction (%)		Unstable angina (%)		Family history of coronary	
	PCI	MIDCAB	PCI	MIDCAB	PCI	MIDCAB	PCI	MIDCAB	PCI	MIDCAB	PCI	MIDCAB
Cisowski [1–3]	NR	NR	26 (52 %)	24 (48 %)	39 (78 %)	38 (76 %)	NR	NR	5 (10 %)	4 (8 %)	20 (40 %)	22 (44 %)
Drenth [3, 5–7]	18	24	30	37	45	41	NR	NR	NR	NR	50	46
Diegeler [8–10]	45	45	25	25	70	73	62 ± 15	63 ± 11	NR	NR	18	17
Reeves [11]	NR	NR	NR	NR	NR	NR	NR	NR	NR	NR	NR	NR
Hong [12]	21.8	22.9	40.3	45.7	54.6	51.4	52.8 ± 8.8	51.9 ± 9.1	50.4	42.9	9.3	10
Kim [13]	22	22	45	55	60	70	51 ± 11	49 ± 13	65	55	NR	NR
Thiele [14]	23	23	14	18	55	55	65 (60–66)	65 (60–70)	NR	NR	NR	NR

### Assessment of mortality

**Fig. 2:**
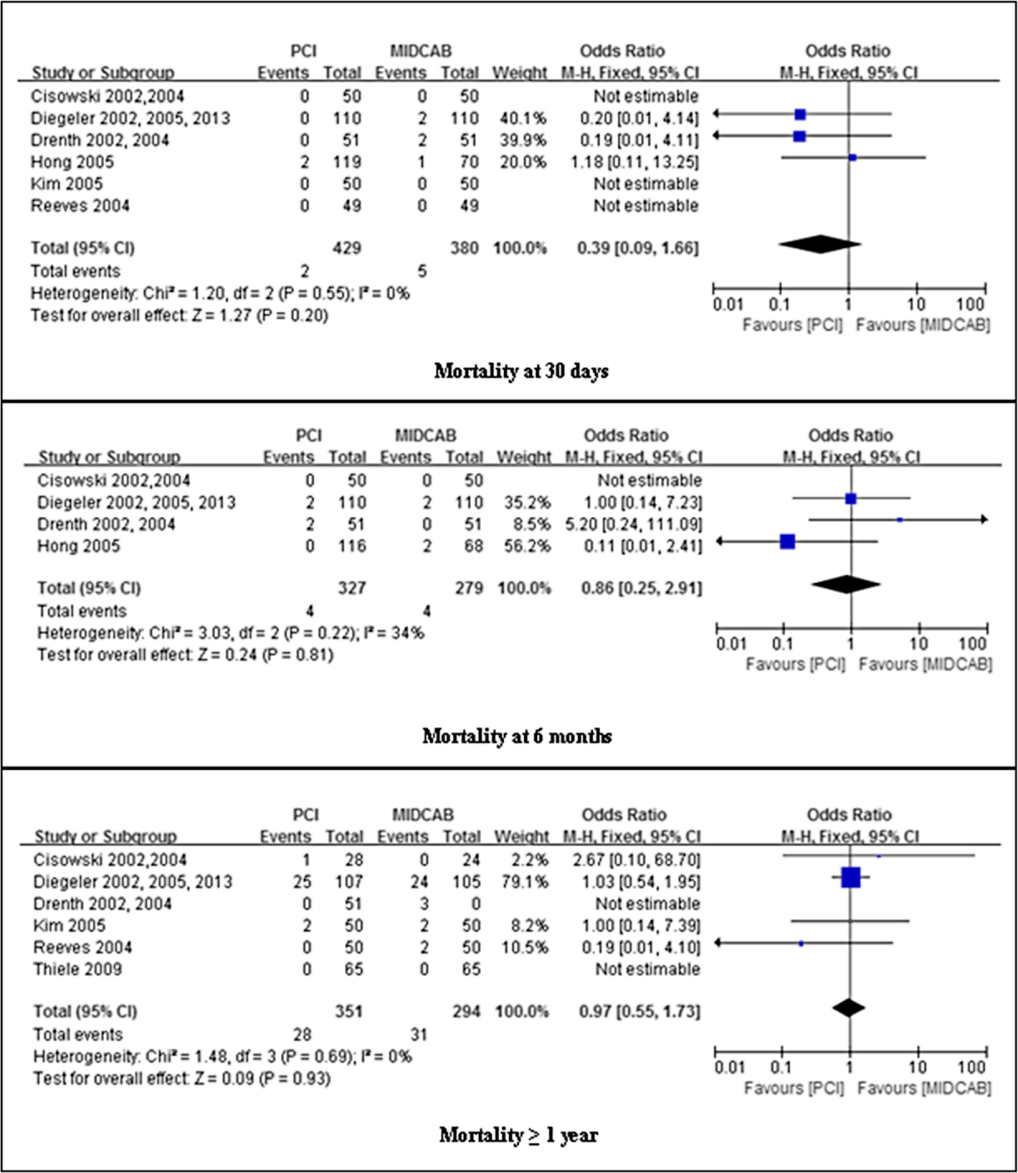
Comparison of PCI versus MIDCAB for the outcome of mortality at 30 days, 6 months, and beyond 1 year follow-up. PCI, percutaneous coronary intervention; MIDCAB, minimally invasive direct coronary artery bypass

As shown in Fig. 2, at 30 days follow-up, PCI and MIDCAB were not different in risk of mortality (0.5 % vs 1.3 %; RR, 0.39; 95 % confidence interval [CI], 0.09-1.66; *P* = 0.20; I^2^ = 0 %). At 6 months follow-up, the overall OR of mortality showed no difference between PCI and MIDCAB (1.2 % vs 1.4 %; OR, 0.86; 95 % CI, 0.25-2.91; *P* = 0.81). We found a moderate level of heterogeneity (I^2^ = 42 %, *p* = 0.22) for the pooled results for mortality. Similarly, at ≥ 1 year follow-up, PCI and MIDCAB were not different in risk of mortality (8.0 % vs 10.5 %; OR, 0.97; 95 % CI, 0.55-1.73; *P* = 0.93; I^2^ = 0 %).

### Assessment of myocardial infarction

**Fig. 3:**
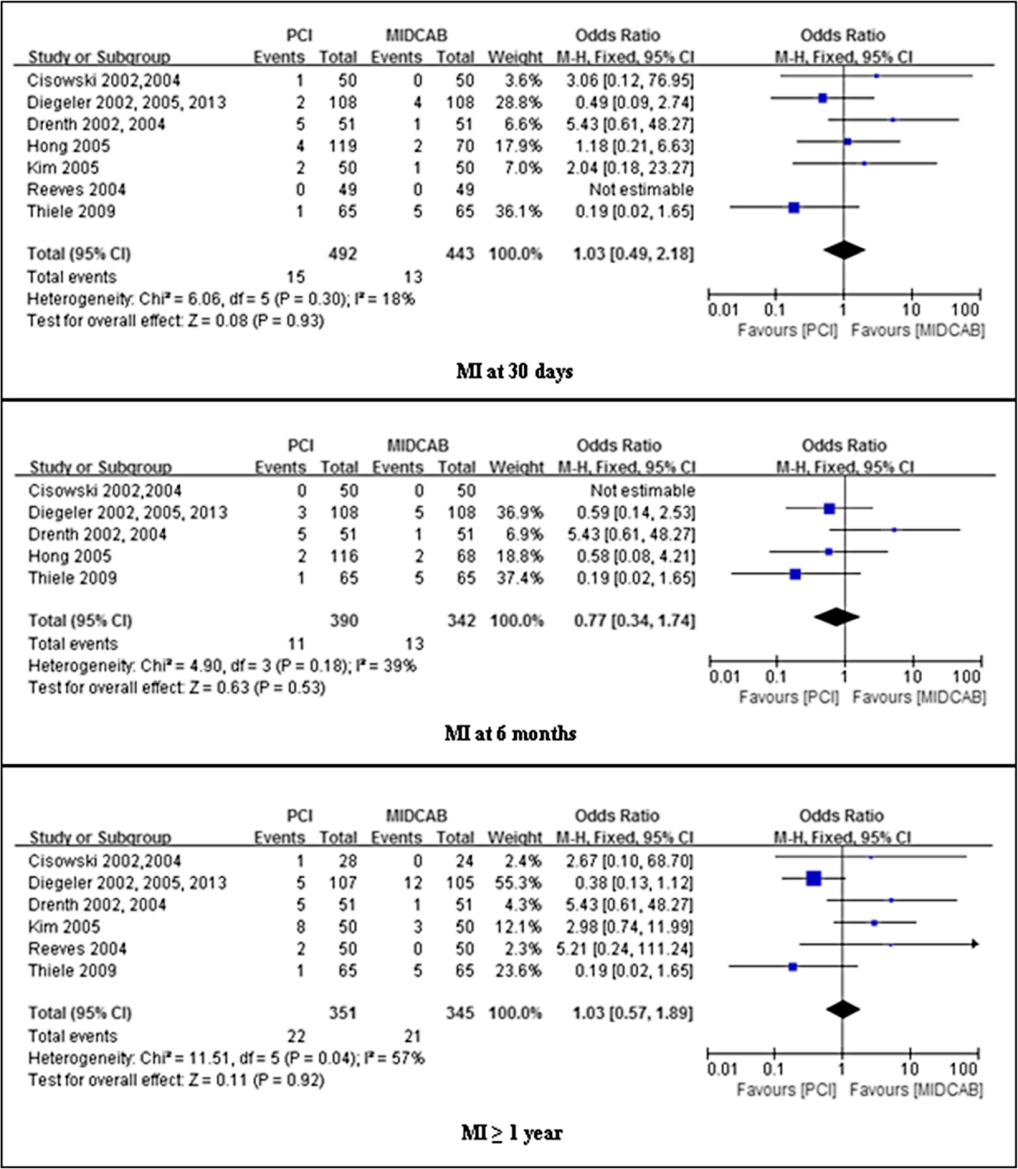
Comparison of PCI versus MIDCAB for the outcome of MI at 30 days, 6 months, and beyond 1 year follow-up. PCI, percutaneous coronary intervention; MIDCAB, minimally invasive direct coronary artery bypass

Myocardial infarction was not significantly different between PCI and MIDCAB at 30 days (3.0 % vs 2.9 %; OR, 1.03; 95 % CI, 0.49-2.18; *P* = 0.93; I^2^ = 18 %), 6 months (2.8 % vs 3.8 %; OR, 0.77; 95 % CI, 0.34-1.74; *P* = 0.53), ≥ 1 year (6.3 % vs 6.1 %; OR, 1.03; 95 % CI, 0.57-1.89; *P* = 0.92). We found a low level of heterogeneity (I^2^ = 39 %, *p* = 0.18) in the pooled results for MI at 6 months and moderate level of heterogeneity (I^2^ = 57 %, *p* = 0.04) at ≥ 1 year (Fig. 3).

### Assessment of TVR

**Fig. 4:**
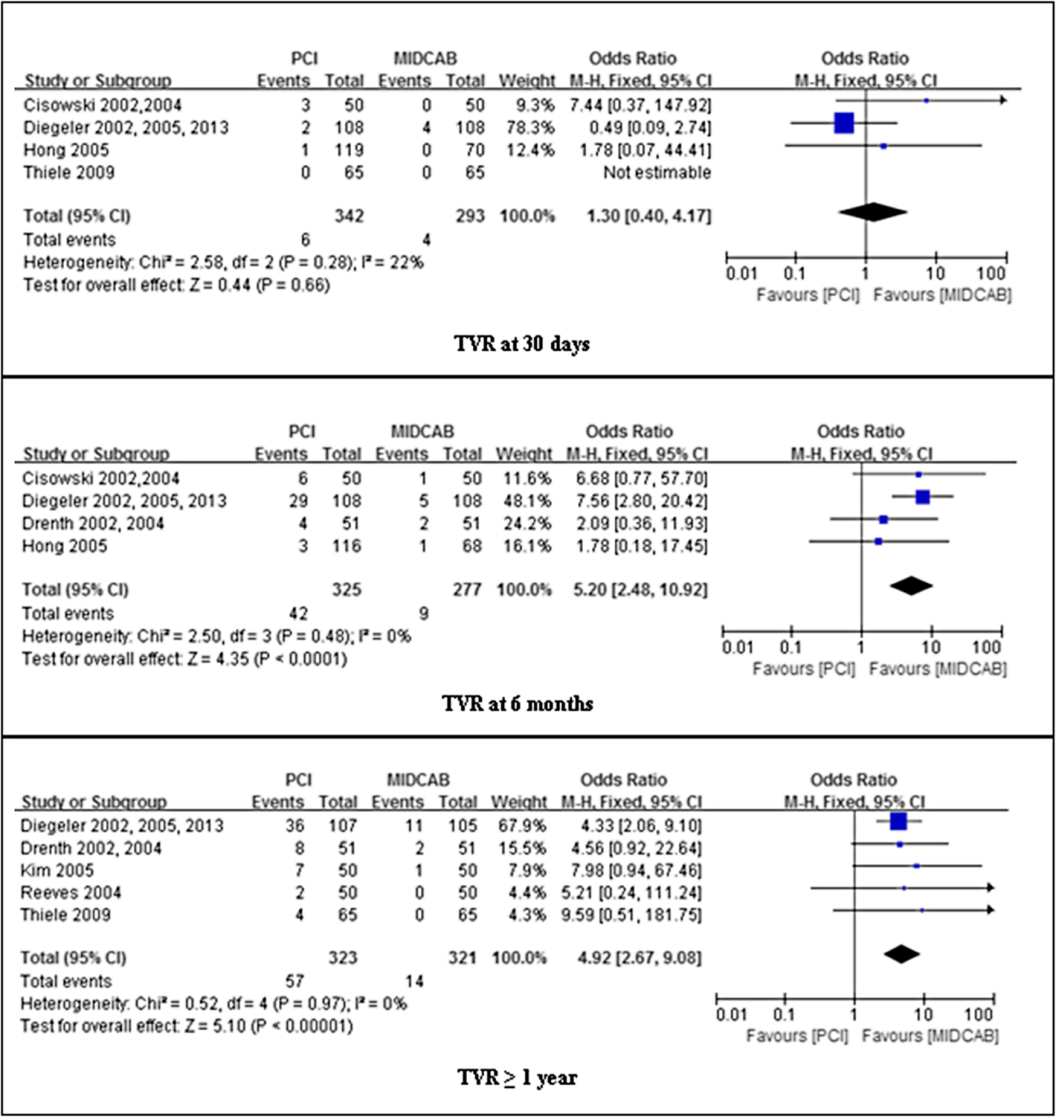
Comparison of PCI versus MIDCAB for the outcome of TVR at 30 days, 6 months, and beyond 1 year follow-up. PCI, percutaneous coronary intervention; MIDCAB, minimally invasive direct coronary artery bypass; TVR, target vessel revascularization

The incidence of TVR was similar in patients undergoing PCI with PCI versus MIDCAB at 30 days (1.75 % vs 1.37 %; OR, 1.30; 95 % CI, 0.40-4.17; *P* = 0.66; I^2^ = 22 %). However, the TVR was significantly higher after PCI compared with MIDCAB at 6 months (12.9 % vs 3.2 %; OR, 5.20; 95 % CI, 2.48-10.92; *P* < 0.001; I^2^ = 0 %), ≥ 1 year (17.6 % vs 4.4 %; OR, 4.92; 95 % CI, 2.67-9.08; *P* <0.00001; I^2^ = 0 %). These results are summarized in Fig. 4.

### Assessment of MACE

**Fig. 5:**
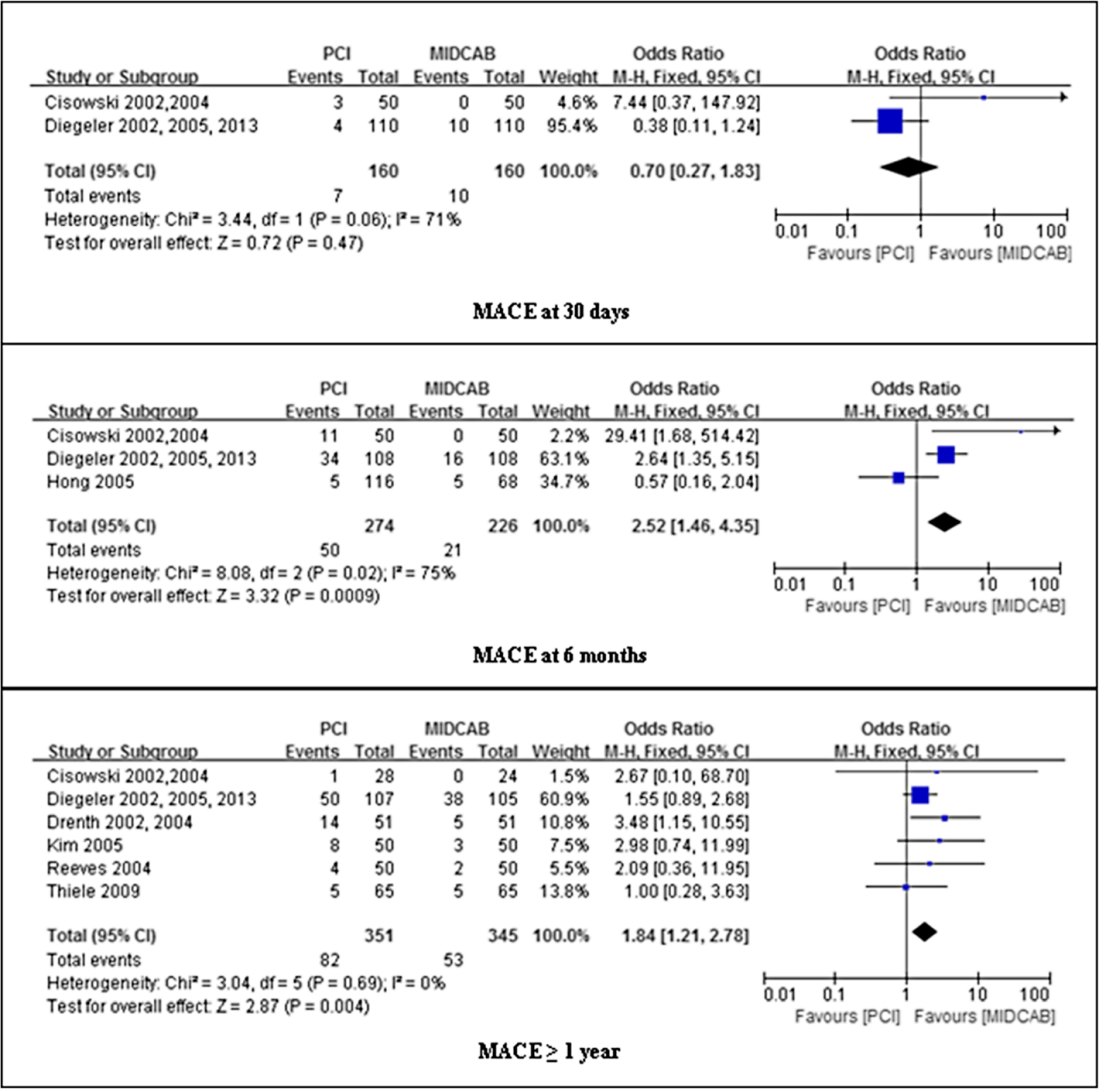
Comparison of PCI versus MIDCAB for the outcome of MACE at 30 days, 6 months, and beyond 1 year follow-up. PCI, percutaneous coronary intervention; MIDCAB, minimally invasive direct coronary artery bypass; MACE, major adverse coronary events

No significant difference was found between DES and CABG groups in the risk of MACE at 30 days follow-up (4.4 % vs 6.3 %; OR, 0.70; 95 % CI, 0.27-1.83; *P* = 0.89; I^2^ = 71 %). MACE, however, occurred significantly more frequently after PCI than MIDCAB at 6 months (18.2 % vs 9.3 %; OR, 2.12; 95 % CI, 1.19-3.79; *P* = 0.0009; I^2^ = 75 %), and ≥ 1 year (23.4 % vs 15.4 %; OR, 1.84; 95 % CI, 1.21-2.78; *P* = 0.004; I^2^ = 0 %). These results are summarized in Fig. 5.

### Assessment of angina recurrence

**Fig. 6:**
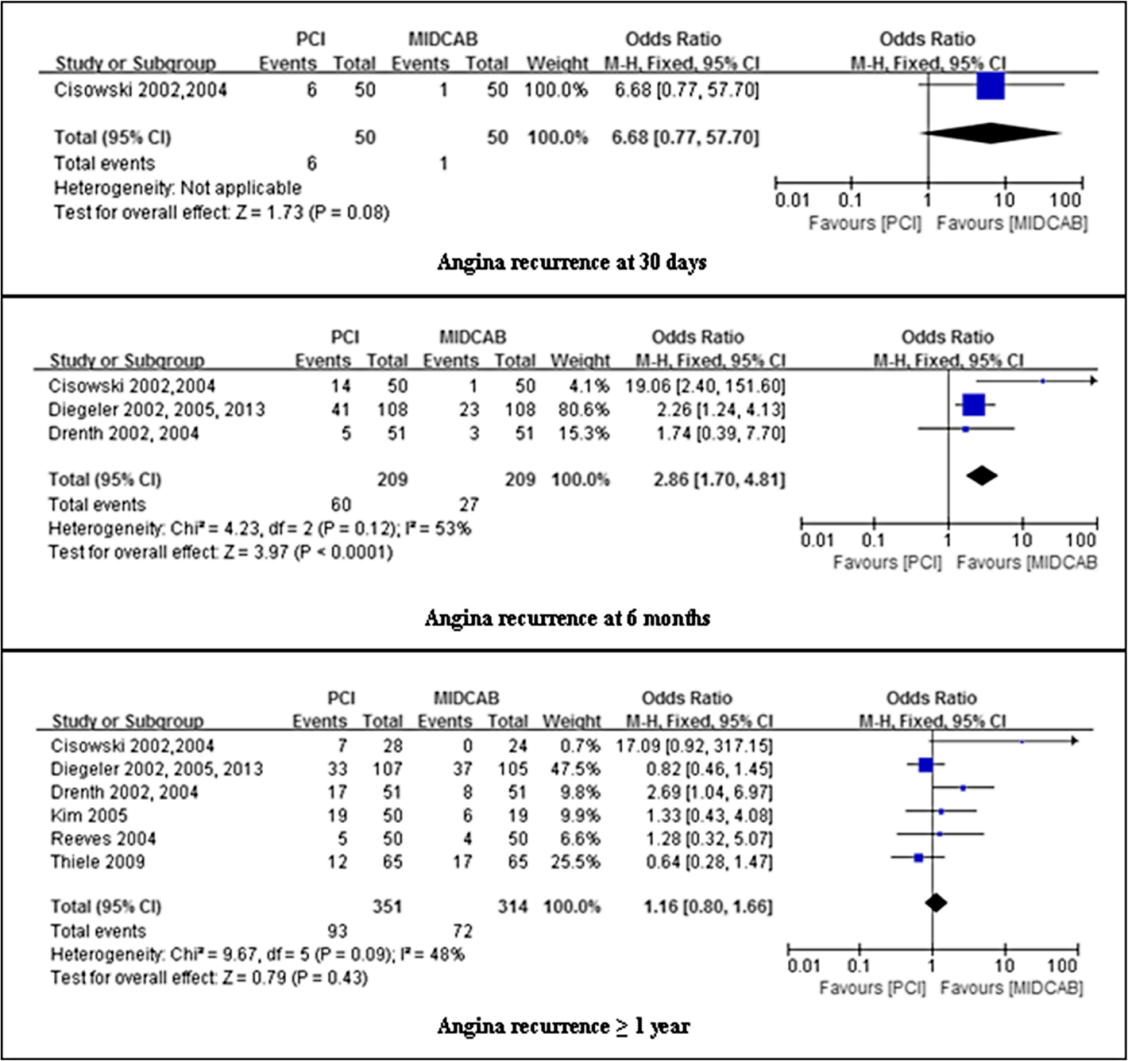
Comparison of PCI versus MIDCAB for the outcome of angina recurrence at 30 days, 6 months, and beyond 1 year follow-up. PCI, percutaneous coronary intervention; MIDCAB, minimally invasive direct coronary artery bypass

Pooled effects showed no significant difference in the incidence of angina recurrence between the PCI group and the MIDCAB group during the initial 30 days (12 % vs 2 %; OR, 6.68; 95 % CI, 0.77-57.70; *P* = 0.08) and over 1 years follow-up (26.5 % vs 23.2 %; OR, 1.16; 95 % CI, 0.80-1.16; *P* = 0.43; I^2^ = 48 %). However, at 6 months follow-up risk for angina recurrence was significantly lower in the MIDCAB group compared to the PCI group (28.7 % vs 12.9 %; OR, 2.86; 95 % CI, 1.70-4.81; *P* < 0.0001). We found a moderate level of heterogeneity (I^2^ = 53 %, *p* = 0.12) for the pooled results for angina recurrence at 6 months follow-up. These results are summarized in Fig. 6.

### Assessment of stroke

**Fig. 7:**
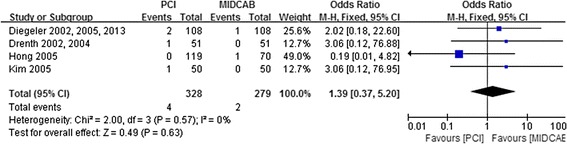
Comparison of PCI versus MIDCAB for the outcome of stroke at 30 days, 6 months, and beyond 1 year follow-up. PCI, percutaneous coronary intervention; MIDCAB, minimally invasive direct coronary artery bypass

Figure 7 shows the overall OR as well as the ORs of individual trials regarding stroke. No heterogeneity across the trials was observed regarding this event (I^2^ = 0 %, *p* = 0.57). There was no significant difference in the risk of stroke between PCI and MIDCAB (1.2 % vs 0.7 %; OR, 1.39; 95 % CI, 0.37-5.20; *P* = 0.63; Fig. 7).

### Assessment of restenosis in target vessel

**Fig. 8:**
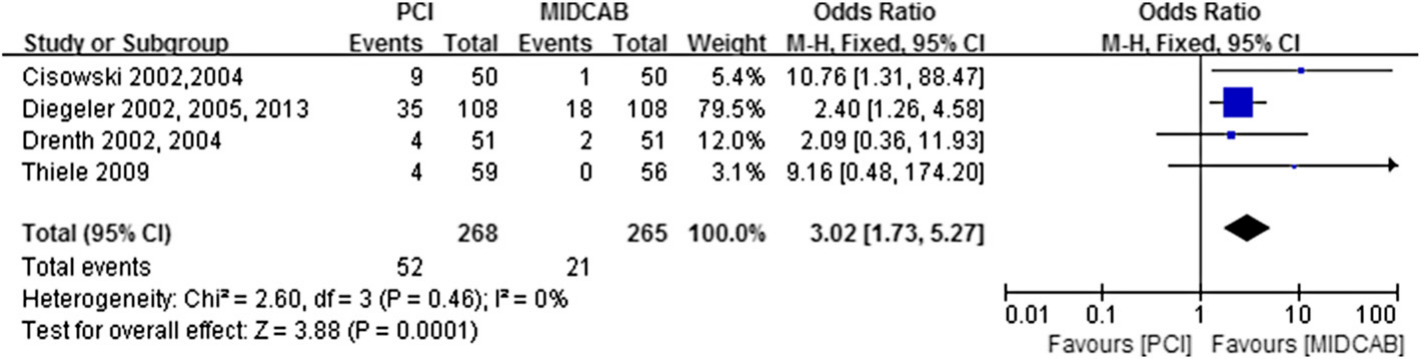
Comparison of PCI versus MIDCAB for the outcome of restenosis in target vessel at 30 days, 6 months, and beyond 1 year follow-up. PCI, percutaneous coronary intervention; MIDCAB, minimally invasive direct coronary artery bypass

Figure 2 shows the overall OR as well as the ORs of individual trials regarding restenosis in target vessel. There was no heterogeneity across the trials. The analysis indicated the risk of restenosis in target vessel was a significantly higher in patients treated with PCI compared with MIDCAB (19.4 % vs 7.9 %; OR, 3.02; 95 % CI, 1.73-5.27; *P* = 0.0001; I^2^ = 0 %; Fig. 8).

## Discussion

This meta-analysis of RCT studies involving patients with single LAD coronary artery disease showed that risk of mortality and myocardial infarction in patients who receiving PCI were not significantly different from those underwent MIDCAB in short-term and long-term follow-ups. However, TVR occurred more frequently in patients treated with PCI compared to those treated with MIDCAB. MACCE occurrence was not different between PCI and MIDCAB in short-term (30 days); however, in long-term (≥1 year follow-up), MACCE occurrence was significantly lower after MIDCAB than after PCI.

Patients with LAD constitute a challenging treatment group, often with significant comorbidities that increase the risk of mortality and healthcare costs. The choice of the appropriate LAD revascularization technique is usually guided by the angiographic characteristics of the stenosis, but also by the respective expertise of the interventional and surgical teams and preferences of the patient. Recent technical progresses in DES have reduced restenosis rates and mortality, repeat revascularization, and MACE compared with bare metal stents [15]. However, the selection for the optimal revascularization procedure remains controversial. In previous meta-analysis found that MIDCAB is associated with lower rates of recurrence of angina, MACCE, and need for repeat revascularization than PCI [16, 17].

Recent a 10-year long-term follow-up data of clinical controlled trials comparisons between PCI and MIDCAB showed that PCI was associated with a statistically significant increase in target vessel revascularization rate [10]. The present meta-analysis, summarizing results from RCT studies including a large ‘real world’ LAD population, supports the conclusion that PCI continues to be associated with a significantly higher risk of TVR and MACE compared with MIDCAB. Our meta-analysis findings need to be confirmed in ongoing, multicentric large clinical trials.

There are several important limitations in this study. First, the benefit of PCI or MIDCAB may depend on the extent and complexity of coronary artery disease. Second, some results of our meta-analysis have significant heterogeneities. Third, definitions of end points were different across included studies. Fourth, we did not have access to further propensity analysis or stratified analysis to better define differences between treatment groups. Fifth, follow-up length for rates of stroke and restenosis was too short to detect differences between the two groups. Further, most of the follow-up in this analysis is up to 1 year. The long-term durability of PCI versus MIDCAB remains undetermined and will require longer follow-up. Finally, we would also like to point out the publication bias exaggerating the positive effects of MIDCAB when meta-analysis was based on previously published studies, due to positive results are more tendency to be published than negative results.

## Conclusion

In conclusion, MIDCAB reduces the need for TVR, and incidence of MACE at 6 months and beyond 1 year when compared with PCI. MIDCAB and PCI were not significantly different on the mortality, incidence of myocardial infarction and troke. Overall, current evidence suggests that MIDCAB is still superior to PCI for the majority of patients with LAD.
